# Deferoxamine synergizes with transforming growth factor-β signaling in chondrogenesis

**DOI:** 10.1590/1678-4685-GMB-2016-0324

**Published:** 2017-08-14

**Authors:** Zheng Huang, Guangxu He, Yanke Huang

**Affiliations:** 1 Department of Spinal Surgery, Shenzhen Nanshan Hospital of Guangdong Medical College, Shenzhen, Guangdong, China; 2 Department of Orthopedics, Second Xiangya Hospital, Central South University, Changsha, Hunan, China; 3 Clinic of Psychology, Shenzhen Nanshan Maternity and Child Healthcare Hospital, Shenzhen, Guangdong, China

**Keywords:** chondrogenesis, deferoxamine, osteoarthritis, TGF-β

## Abstract

Osteoarthritis, also known as degenerative arthritis or degenerative joint disease, is an epidemic disease that affects millions of people worldwide. Despite extensive recent work on the cellular biology of osteoarthritis, the precise mechanisms involved are still poorly understood and there is no effective treatment for this disease. The role of transforming growth factor-beta (TGF-β) in promoting chondrogenesis and inducing the expression of cartilage-specific extracellular matrix molecules to form cartilage is well-established. Historically, TGF-β has been considered to prevent osteoarthritis, but recent work suggests that TGF-β overexpression accelerates the progression of osteoarthritis *in vivo*. Clinically, it is therefore important to limit TGF-β expression while still providing effective treatment of osteoarthritis. One possible approach to achieve this effect would be to use a combination of TGF-β with other small molecular chemical compounds. Hypoxia promotes chondrogenesis and the usefulness of deferoxamine, a chelating agent that mimics hypoxia, in stimulating chondrogenesis has been investigated in clinical trials. In this study, we investigated the role of deferoxamine in TGF-β-induced chondrogenesis in pre-chondrogenic cells and examined whether deferoxamine synergizes with the TGF-β signaling pathway to promote chondrocyte differentiation.

Osteoarthritis (OA) is a degenerative disease of articular cartilage that is projected to affect > 50 million people in the United States by the year 2020 (Wang *et al.*, 2011). OA is widely considered to be a joint disorder, the key pathological feature of which is cartilage destruction (Bijlsma *et al.*, 2011). In the last several decades, numerous studies have examined the pathogenic mechanisms of OA, but a full understanding of the initial factors involved in the development of OA remains to be achieved. The recent tremendous advances in genome-wide association analysis have revealed a correlation between genetic variants of the TGF-β signaling pathway components (from ligands to transcription factors) and the occurrence of clinical OA (Yamada *et al.*, 2000; Lau *et al.*, 2004).

The TGF-β family is a large family of growth factors with an indispensable role in embryonic development, postnatal homeostasis and the regulation of cell proliferation, differentiation, apoptosis and migration in different tissues or cell types. The TGF-β signaling pathway stimulates chondrogenesis in bone marrow stromal cells and mesenchymal stem cells (Hanada *et al.*, 2001; Oka *et al.*, 2007; Shen *et al.*, 2014). In recent years, there has been increasing attention to the role of TGF-β in OA in murine models and humans (Kizawa *et al.*, 2005). Since the induction of chondrogenesis via mesenchymal stem cells or pre-chondrocytes is an important phenomenon in delaying the progression of OA then interference with the action of TGF-β could be a useful therapeutic approach for treating OA.

Multiple intra-articular injections of TGF-β can alter articular cartilage and surrounding tissues to produce features that strongly resemble those of experimental and spontaneous OA in mice; these findings indicate that high levels of TGF-β can accelerate the onset and progression of OA *in vivo* (van Beuningen *et al.*, 2000). Hence, in clinical trials, it is critical to control the dose of TGF-β while retaining the efficiency in inhibiting OA. To achieve this goal, the cross-talk between TGF-β and other molecular pathways need to be urgently studied.

Hypoxia has recently emerged as a key factor that facilitates chondrogenesis in mesenchymal stem cells (Zhong *et al.*, 2015). Hypoxia enhances the expression and activity of HIF-1α, a key component in the genetic program that regulates chondrogenesis by modulating SOX9 expression in hypoxic pre-chondrogenic cells during early skeletogenesis (Amarilio *et al.*, 2007). Deferoxamine (DFO) has medical applications as a chelating agent used to remove excess iron from the body (Ramurthy and Miller, 1996) and has been approved by the Federal Drug Administration to treat chronic iron overload and acute iron intoxication. DFO acts by binding free iron in the bloodstream and enhancing its elimination in the urine. By removing excess iron, DFO reduces the damage to various organs and tissues, such as the liver. In this study, we examined the usefulness of a combination of DFO and TGF-β for treating bone marrow stromal cells and sought to determine whether DFO synergizes with TGF-β in inducing chondrogenesis. Our findings suggest that a combination of these two substances could be useful for treating OA.

The chondrogenic cell line ATDC5 was purchased from the American Tissue Culture Collection (ATCC, Manassas, VA, USA) and cultured in DMEM: Ham F12 (1:1, v/v) medium supplemented with 2 mM glutamine and 10% fetal bovine serum (FBS). ATDC5 cells were incubated in a 5% CO_2_ atmosphere at 37 °C and sub-cultured when they reached 70-80% confluence. The cells were seeded at a density of 1x10^3^ per cm^2^ in 6-well plates for two days and then treated with or without 100 μM deferoxamine mesylate (DFO; Sigma Chemical Co., St. Louis, MO, USA) or human recombinant transforming growth factor-β1 protein (TGF-β1, 10 ng/mL; R&D Systems, Minneapolis, MN, USA) for the indicated number of days.

Primary cultures of chondrocytes were isolated from the ventral half of the rib cage of embryos, as previously described (Lefebvre *et al.*, 1994). Briefly, rib cages and sterna were dissected from E18.5 embryos, rinsed in phosphate-buffered saline (PBS) and incubated at 37 °C for 1 h in PBS containing pronase E (2 mg/mL; Sigma), rinsed again with PBS and then incubated with bacterial collagenase D (3 mg/mL; Sigma) in DMEM at 37 °C in a 7.5% CO_2_ atmosphere for 1 h until the soft tissue detached from the cartilage after gentle titration. The cartilage was then further digested with collagenase D for 2 h. Residual bony parts were discarded. The remaining cell suspension was filtered through a 70-μm cell strainer (Becton Dickinson) and rinsed in αMEM containing 10% FBS. The isolated chondrocytes were seeded at a density of 1 x 10^5^ cells/cm^2^ into 6-well plates in αMEM containing 10% FBS and were cultured at 37 °C in a 5% CO_2_ atmosphere.

A modified micromass culture system was used, as previously described (Shi *et al.*, 2012). Briefly, the cells were harvested and resuspended in chondrogenic medium at 1 x 10^7^ cells/mL. Twenty microliters of cell suspension was carefully placed in each well of a 24-well plate. The cells were allowed to adhere at 37 °C in a 5% CO_2_ atmosphere for 2 h followed by the addition of 500 μL of growth medium supplemented with TGF-β1 (10 ng/mL). After 24 h, the cell droplets coalesced and became spherical. The culture medium was changed every three days and after seven days the micromass was stained with Alcian blue (Sigma). Briefly, cells were fixed with 0.1% glutaraldehyde in PBS for 20 min at room temperature and stained in Alcian blue solution for 1 h and then rinsed with 0.1 M HCl.

ATDC5 cells and primary chondrocytes were seeded on six-well plates and allowed to reach ~80% confluency. Cell viability and proliferation were assessed with the AlamarBlue^®^ assay (Invitrogen), according to the manufacturer's instructions. The cells were incubated in medium supplemented with 10% (v/v) AlamarBlue^®^ fluorescent dye for 2 h after treatment for three days at 37 °C in a 5% CO_2_ atmosphere. One hundred microliter aliquots of medium were then transferred to 96-well plates (Corning, Corning, NY, USA) and the absorbance was read at 570 nm and 590 nm in a Multiscan UV visible spectrophotometer (Safire2; TECAN, Mannedorf, Switzerland). Unseeded 96-well plates with the same medium were used as blanks. The results were expressed as a percentage relative to the non-treated (control) group (NC).

Cells were harvested after the indicated treatment and total RNA was isolated from whole cells with a QIAGEN RNeasy kit (74104, QIAGEN, Germantown, MD, USA) and was transcribed into cDNA by using iScript cDNA synthesis kit (BioRad, Hercules, CA, USA). Fast-start SYBR Green (BioRad) and 0.1 mM of primers were used in each reaction. Ribosomal 18S RNA was used for normalization. PCR was done with a Step-One machine (Applied Biosystems, Foster City, CA, USA). The primer sequences used were: 18S (Forward: CGGCTACCACATCCAAGGA; Reverse: GCTGGAATTACCGCGGCT), Sox9 (Forward: AGTACCCGCATCTGCACAAC; Reverse: ACGAAGGGTCTCTTCTCGCT), collagen type 2 (Col2a1) (Forward: GGGTCACAGAGGTTACCCAG; Reverse: ACCAGGGGAACCACTCTCAC) aggrecan (Forward: CCCAGGATAAAACCAGGCAG; Reverse: CGGCCAAGGGTTGTAAATGG) and Ocn (Bglap) (Forward: CAGCGGCCCTGAGTCTGA; Reverse: GCCGGAGTCTGTTCACTACCTTA). In all cases, the fold change in expression was calculated using the Ct (2^-ΔΔCt^) comparative method (Shi *et al.*, 2011).

Cells were lysed on ice for 30 min in lysis buffer containing 50 mM Tris-HCl, pH 7.4, 150 mM NaCl, 1% Nonidet P-40 and 0.1% SDS supplemented with protease inhibitors (10 mg/mL leupeptin, 10 mg/mL pepstatin A, and 10 mg/mL aprotinin). For western blotting, 15 μg samples were run by SDS-PAGE on 12% polyacrylamide gels and electrotransferred to nitrocellulose membranes (Whatman, Piscataway, NJ, USA). The primary antibody used was HIF1α (3716, Cell Signaling Technology, Inc., Danvers, MA, USA) at a dilution of 1:1,000. Protein loading was normalized to GAPDH (Cell Signaling Technology, Inc.) detected with antibody was used at a 1:2,000 dilution. HRP-conjugated secondary antibody was used at a 1:2,000 dilution. The antigen-antibody complexes were visualized using an enhanced chemiluminescence detection system (Millipore, Billerica, MA, USA), as recommended by the manufacturer. The immunoreactive bands were assessed quantitatively in triplicate by normalizing the band intensities to their respective controls on films that were scanned and analyzed with Alpha Image software.

Quantitative data were expressed as the mean ± SD. Statistical comparisons between the control and treatment groups were done using ANOVA, followed by the Student-Newman-Keuls post-hoc test. All the data analyses were done using SPSS software v.16.0 (SPSS Inc, Chicago, IL, USA). A value of p < 0.05 indicated significance.

We initially determined the concentration of TGF-β1 by checking Sox9 expression. As shown in Figure 1A, 10 ng/mL was the lowest concentration to induce gene expression and this concentration was therefore used in subsequent experiments. ATDC5 cells were treated with TGF-β1 (10 ng/mL), 100 μM DFO or both for three days and subsequent qPCR showed that, as expected, there was a significant increase in the expression of the Sox9 (Figure 1B), collagen type 2 (Col2a1) (Figure 1C) and aggrecan (Figure 1D) genes in response to TGF-β1. Interestingly, DFO alone did not alter the expression of these genes, but increased that of Sox9 from six-fold to around ten-fold in the presence of TGF-β1 (Figure 1B). When incubated with TGF-β1, DFO increased Col2a1 expression from seven-fold to 13-fold (Figure 1C) and that of aggrecan from five-fold to 12-fold (Figure 1D). There was no significant alteration in the expression of the osteogenic differentiation marker Ocn (Figure 1E).

**Figure 1:**
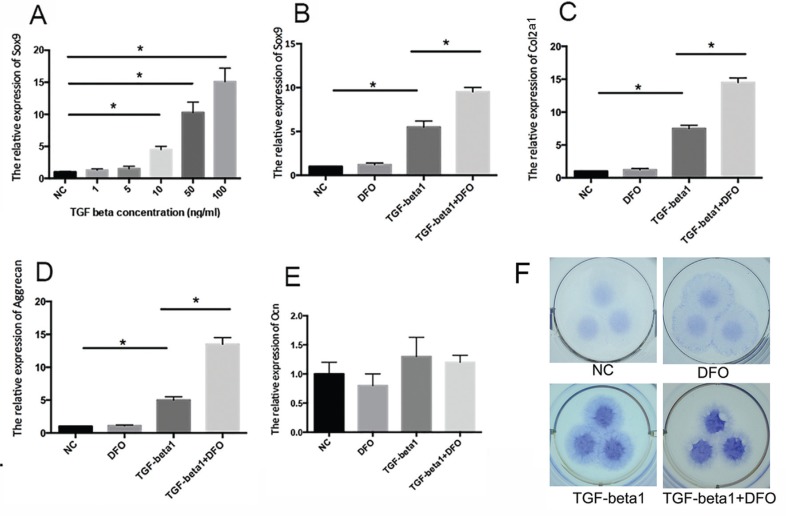
DFO synergizes with TGF-β1 in chondrogenesis in ATDC5 cells. (A) Sox9 gene expression in ATDC5 cells treated with different concentrations of TGF-β1. NC – non-treated control cells. *p < 0.05 compared to NC cells. (B-E) ATDC5 cells were treated with DFO, TGF-β1 or both for three days, after which Sox9 (B), Col2a1 (C), aggrecan (D) and Ocn (E) gene expression was assessed. In panels A-E, the columns represent the mean + 1 SD (n = 3 each). *p < 0.05 for cells treated with TGF-β1 *vs.* NC cells and for cells treated with TGF-β1 *vs.* cells treated with TGF-β1 + DFO. (F) ATDC5 cells were treated with DFO, TGF-β1 or both for seven days in micromass culture. The cells were then fixed and stained with Alcian blue. This assay was done in triplicate.

Treatment with TGF-β1 enhanced the Alcian blue staining in ATDC5 micromass culture. In contrast to the changes in gene expression, DFO alone only slightly increased this staining, possibly because hypoxia not only induced the expression of chrondrogenesis-related genes but also inhibited matrix degradation. DFO significantly darkened the staining in the presence of TGF-β1 indicating that the combination of these stimuli resulted in more collagen matrix accumulation (Figure 1F).

TGF-β1 increased cell proliferation by two-fold compared to the non-treated controls, whereas TGF-β1 combined with DFO increased proliferation by five-fold (Figure 2). To confirm this finding of synergism, we repeated the test with primary chondrocytes. Treatment of primary chondrocytes with TGF-β1 (10 ng/mL), 100 μM DFO or both compounds for three days followed by qPCR showed that expression of the Sox9 (Figure 3A), collagen type 2 (Col2a1) (Figure 3B) and aggrecan (Figure 3C) genes was significantly increased after exposure to the combination of DFO and TGF-β1 compared to TGF-β1 alone. This finding was consistent with that for the ATDC5 cell line. There was no change in the expression of the osteogenic gene Ocn in response to DFO and TGF-β1 (Figure 3D). Moreover, DFO was only able to increase cell proliferation in the presence of TGF-β1 and fold changes were dramatically greater than with TGF-β1 alone (Figure 3E). Together, our data indicate that DFO synergized with TGF-β1 in inducing chondrogenesis.

**Figure 2:**
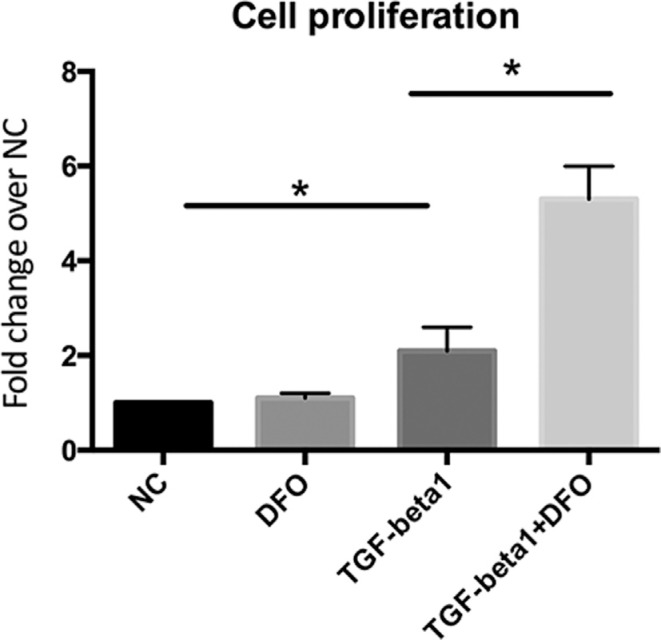
DFO synergizes with TGF-β1 in the proliferation of ATDC5 cells. The cells were treated with DFO, TGF-β1 or both for three days, after which cell proliferation was assessed using the AlamarBlue^®^ assay. The columns represent the mean + 1 SD of the fold-change in cellular proliferation relative to non-treated control cells (NC) (n = 3 each). *p < 0.05 for cells treated with TGF-β1 *vs.* NC cells and for cells treated with TGF-β1 *vs.* cells treated with TGF-β1 + DFO.

**Figure 3:**
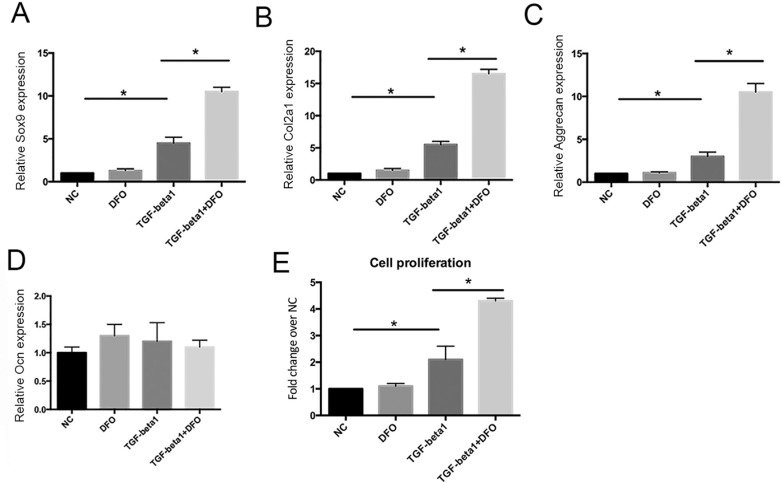
DFO synergizes with TGF-β1 in chondrogenesis and proliferation in primary chondrocytes. (A-D) Primary chondrocytes were treated with DFO, TGF-β1 or both for three days, after which Sox9 (A), Col2a1 (B), aggrecan (C) and Ocn (D) gene expression was assessed. (E) Primary chondrocytes were treated with DFO, TGF-β1 or both for three days, after which cell proliferation was determined by the AlamarBlue^®^ assay. The results are expressed as the fold-change in proliferation relative to non-treated control cells (NC). The columns represent the mean + 1 SD (n = 3 each). *p < 0.05 for cells treated with TGF-β1 *vs.* NC cells and for cells treated with TGF-β1 *vs.* cells treated with TGF-β1 + DFO.

To determine the mechanism by which DFO synergizes with TGF-β1 signaling, we used western blotting to examine expression of the important hypoxia mediator HIF-1α. ATDC5 cells were treated with TGF-β1 (10 ng/mL), 100 μM DFO or both compounds for 1 h and the cellular protein were extracted for western blotting. Treatment with DFO significantly increased HIF-1α protein expression in response to TGF-β1 (Figure 4), which suggested that an increase in hypoxia could be involved in the synergistic effect observed.

**Figure 4:**
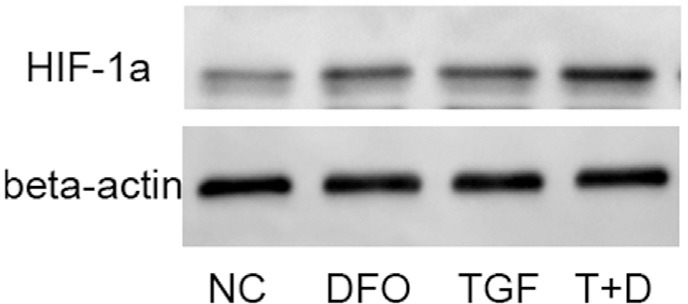
HIF-1α mediates the synergistic effect of DFO in TGF-β1-triggered chondrogenesis in ATDC5 cells. The cells were treated with TGF-β1 (TGF or T; 10 ng/mL), 100 μM DFO (D) or both for 1 h and then lysed to obtain cytosolic proteins for western blotting. β-Actin was used as an internal control (housekeeping protein). NC – non-treated control cells.

Osteoarthritis is a degenerative joint disease that affects the whole joint structure, including articular cartilage and synovial tissue. There is no effective medical therapy for OA, partly because of its poorly understood pathogenesis. The importance of the TGF-β1 pathway in attenuating OA was previously thought to be well-established, but an increasing number of reports have demonstrated that TGF-β1 may also accelerate OA progression through its role in the degradation of subchondral bone and articular cartilage (Xie *et al.*, 2016). Indeed, the dose of TGF-β1 can influence the outcome of OA treatment. These findings indicate that TGF-β1 has additional roles that go beyond its ability to induce chondrogenesis, although the stimulation of chondrogenesis in articular cartilage is still the conventional way for treating OA.

The OA epidemic requires comprehensive solutions, including novel pharmacological strategies. As shown here, DFO, which mimics hypoxia by inducing HIF1α expression, facilitates chondrogenic differentiation and can synergize with TGF-β1 in chondrogenesis. These data suggest that combining the TGF-β1 signaling pathway with hypoxia could provide a potentially more effective means for treating OA.
